# Response of chlorophyll *a* to total nitrogen and total phosphorus concentrations in lotic ecosystems: a systematic review protocol

**DOI:** 10.1186/s13750-017-0097-8

**Published:** 2017

**Authors:** Micah G. Bennett, Kate A. Schofield, Sylvia S. Lee, Susan B. Norton

**Affiliations:** National Center for Environmental Assessment, Office of Research and Development, U.S. Environmental Protection Agency, 1200 Pennsylvania Ave. NW (8623-P), Washington, DC 20460, USA

**Keywords:** Nutrients, Pollution, Water quality, Stressor-response, Stream, River, Primary production, Eutrophication

## Abstract

**Background:**

Eutrophication of freshwater ecosystems resulting from nitrogen and phosphorus pollution is a major stressor across the globe. Despite recognition by scientists and stakeholders of the problems of nutrient pollution, rigorous synthesis of scientific evidence is still needed to inform nutrient-related management decisions, especially in streams and rivers. Nutrient stressor-response relationships are complicated by multiple interacting environmental factors, complex and indirect causal pathways involving diverse biotic assemblages and food web compartments, legacy (historic) nutrient sources such as agricultural sediments, and the naturally high spatiotemporal variabilityof lotic ecosystems. Determining nutrient levels at which ecosystems are affected is a critical first step for identifying, managing, and restoring aquatic resources impaired by eutrophication and maintaining currently unimpaired resources. The systematic review outlined in this protocol will compile and synthesize literature on the response of chlorophyll *a* to nutrients in streams, providing a state-of-the-science body of evidence to assess nutrient impacts to one of the most widely-used measures of eutrophication. This review will address two questions: “*What is the response of chlorophyll* a to *total nitrogen and total phosphorus concentrations in lotic ecosystems?”* and *“How are these relationships affected by other factors?”*

**Methods:**

Searches for published and unpublished articles (peer-reviewed and non-peer-reviewed) will be conducted using bibliographic databases and search engines. Searches will be supplemented with bibliography searches and requests for material from the scientific and management community. Articles will be screened for relevance at the title/abstract and full text levels using pre-determined inclusion criteria; 10% (minimum 50, maximum 200) of screened papers will be examined by multiple reviewers to ensure consistent application of criteria. Study risk of bias will be evaluated using a questionnaire developed from existing frameworks and tailored to the specific study types this review will encounter. Results will be synthesized using meta-analysis of correlation coefficients, as well as narrative and tabular summaries, and will focus on the shape, direction, strength, and variability of available nutrient-chlorophyll relationships. Sensitivity analysis and meta-regression will be used to evaluate potential effects of study quality and modifying factors on nutrient-chlorophyll relationships.

## Background

Nutrient pollution by nitrogen (N) and phosphorus (P)—defined here as nutrient concentrations higher than background or natural levels—is a major stressor of freshwater ecosystems, both across the United States and globally [1–6]. Nutrients and resulting stressors (e.g. oxygen depletion) degrade ecosystem services worth more than $2.2 billion annually in the United States alone [7]. Despite recognition by scientists and stake-holders that nutrient pollution and resulting eutrophication (increased ecosystem metabolism) are problems in fresh waters [1, 4, 5, 8, 9], rigorous synthesis of scientific evidence is still needed to inform nutrient-related management decisions and policies, particularly in streams and rivers [10]. There are several factors that complicate nutrient stressor-response relationships in lotic systems. Several potential nutrient constituents (e.g. nitrate, ammonia) can act as stressors. Causal pathways between nutrients stressors and biological effects are complex and include many indirect effects. These pathways also involve diverse assemblages (e.g. algae, macroinvertebrates, fishes) and food web compartments (e.g. “green” pathways involving primary producers, “brown” pathways involving heterotrophic bacteria and fungi [11, 12]); and many interacting environmental factors are also involved, such as land use, flooding, and stream size, affect stressor-response relationships [13–15]. Temporal factors also complicate relationships, with legacy (historic) nutrient sources contributing to stressors [3, 16, 17]. Finally, high spatiotemporal variability of both nutrient concentrations [18] and lotic systems more generally [19] can complicate evaluation of stressor-response relationships in these systems. The effects of nutrient increases on biota have been documented in streams and rivers with a variety of biological, chemical, and physical conditions; however, to our knowledge, a synthesis of links between nutrient increases and impacts on stream biota that also addresses the influence of differing conditions across a breadth of lotic systems is lacking [20].

Biota integrate impacts over time and so can better represent ecological condition compared to snapshot water quality measurements [21–24]. Environmental managers often use this biological information to evaluate impacts of chronic pollution (e.g. [25]). However, high spatiotemporal variability and other factors (e.g. those mentioned above) can mask links between nutrients and biota [26]. A synthesis of nutrient stressor-response relationships and how these relationships are modified by other factors could aid the setting of regulatory limits and identification impacted systems based on biota (e.g. [27]).

Algae are the main primary producers in lotic systems, and algal biomass is expected to be one of the first ecological endpoints to respond to nutrient pollution [28]. Increases in algal biomass are also associated with many of the negative human health and ecological consequences of eutrophication, such as reduced drinking water quality [29, 30] and altered species composition [4]. Chlorophyll *a* (chl-a) is a photosynthetic pigment used to measure algal biomass [31]. In streams and rivers, researchers may sample benthic chl-a from hard substrates or sestonic chl-a from the water column [31, 32] to determine chl-a concentrations.

This systematic review will compile and synthesize literature on chl-a responses to nutrients in streams and rivers, to provide a state-of-the-science body of evidence for assessing nutrient impacts. The review focuses on total nitrogen (TN) and total phosphorus (TP) concentrations in the water column. These constituents were selected for both ecological and practical reasons. Although dissolved nutrient forms may be more available for immediate uptake by biota, total nutrient forms are often more highly correlated with chl-a [28]. Dissolved forms may undergo rapid uptake and release by primary producers, such that concentrations of dissolved nutrients in the water column may not represent true availability [33, 34]. In contrast, total nutrient forms may best represent trophic state and nutrient limitation in most lotic ecosystems because TN and TP account for N and P held within algae and sediment particles and thus represent integrated measures of biologically available nutrients [26, 34, 35]. TN and TP are also the most common nutrient measures used by environmental managers in the United States and around the globe to assess eutrophication of lotic ecosystems [36].

This review was motivated by a need for comprehensive information on stressor-response relationships to aid water quality scientists at the U.S. Environmental Protection Agency (USEPA) and state environmental agencies in better understanding the effects of nutrient pollution. In several meetings held during 2016–2017, these potential end users helped refine the scope, specific questions and objectives (including the relevant population, exposure, and outcome) of the systematic review, and the modifying factors of interest.

## Objective of the review

The primary question addressed by this review is: *What is the response of chl*-*a to TN and TP concentrations in lotic ecosystems?* The nutrient stressor (TN or TP) and biotic response (chl-a) were chosen based on measures commonly used by U.S. state agencies to evaluate and make regulatory decisions about impairment of lotic ecosystems due to eutrophication. This question consists of the following components:

### Population:

Lotic fresh waters, or mesocosms that mimic these systems, in any geographic location.

### Exposure:

Concentration of TN or TP. We define TN as the sum of ammonia N, nitrate N, nitrite N, and organic nitrogen forms; we define TP as the sum of dissolved and particulate phosphorus forms.

### Comparator:

Control group (no added TN or TP, or low exposure to TN or TP) (for experimental studies), or comparison to lower or higher TN or TP concentrations across a gradient (for observational studies).

### Outcome:

Chl-a concentration (sestonic or benthic).

The secondary question addressed by this review is: *How are the relationships identified in the primary question affected by other factors?* An initial list of potential modifying factors is provided below (see “Methods” and “Potential effect modifiers and reasons for heterogeneity”); others may be added as studies are examined in more detail.

## Methods

### Search strategy

#### Search terms and filters

Bibliographic databases will be searched using a combination of terms representing the nutrient stressors (TN or TP), the biological response (chl-a), and habitat- or study-specific terms (e.g. terms associated with types of lotic fresh waters and experimental stream studies) (Table 1). Databases vary in how they handle search strings, so searches will be adapted as needed for each search. An appendix of search strings used for each database will be provided in the full systematic review (see Additional file 1 for an example based on the Web of Science^™^ database). Books, book chapters, pamphlets and conference abstracts will be excluded from consideration unless they are submitted through calls for additional information (see “Supplemental searches”), because they generally do not have sufficient relevant primary data and results to extract, and non-electronic library resource limitations prevent a full evaluation of these resources. No language restrictions will be applied to database searches, and any other filters used for specific databases (e.g. excluding full text search to limit irrelevant literature) will be detailed in the full systematic review.

#### Databases

At least 16 bibliographic databases, representing peer-reviewed, non-peer-reviewed, and unpublished material, will be searched to obtain articles for the review (Table 2). When databases limit the search results that can be viewed or downloaded, results will be filtered by year, when possible, to obtain subsets for viewing and download. Due to limitations on batch downloading of citations, three databases (DART, National Technical Reports Library, and OpenGrey) will be treated similarly to website searches and the first 50 items returned (for separate searches for TN and TP) will be examined (see below) (Table 2).

#### Specialist websites

Websites of the following organizations will be searched for relevant literature:

- U.S. Environmental Protection Agency
- U.S. state- and territory-level environmental agency websites (56 total entities)
- U.S. Department of Agriculture
- U.S. Forest Service
- U.S. Fish and Wildlife Service
- U.S. Geological Survey
- U.S. National Oceanic and Atmospheric Administration (NOAA)
- NOAA Fisheries
- National Park Service
- World Wildlife Fund
- American Rivers
- International Rivers
- The Nature Conservancy
- United Nations Environment Program
- European Environment Agency
- European Commission Joint Research Center
- Environment and Climate Change Canada (http:ec.gc.ca/default.asp?lang=En&n=FD9B0E51-1)
- Fisheries and Oceans Canada
- Canadian Council of Ministers of the Environment

The first 50 items returned, sorted by relevance, will be examined for each search. For websites without a search function, relevant “publications” sections will be examined to find documents. Because many websites do not accept Boolean search strings, separate searches will be conducted for TN and TP, and a smaller set of terms will be used each of these searches. All website searches will be documented in a spreadsheet that will include the search date, the specific web URL and search terms used for each site, any website sub-sections used, the total number of items returned, and the number of items deemed relevant. Although the specialist website list is biased toward western countries, resource constraints limit our ability to search more broadly in non-English speaking countries. The “Supplemental searches” will be used to increase capture of relevant articles from other countries.

#### Search engines

Searches using Google and Google Scholar will be conducted, and the first 50 search results will be examined for relevance as with website searches. Separate searches will be conducted for TN and TP, and search terms used for each search will be documented.

#### Supplemental searches

To supplement these searches, additional resources will be requested from colleagues with disciplinary knowledge and through ECOLOG-L, Twitter, and ResearchGate. “Snowball” searches will also be conducted: references that cite or are cited by a small set of highly relevant literature (see below) will be compiled and any novel references not found during database searches will be evaluated.

#### Reference management

Articles returned by the search strategy will be stored in an EndNote library. Duplicate entries will be removed, and an initial title screen within EndNote will be used to remove entries that are clearly not relevant (e.g. Front Matter, Meeting Programs and Abstracts, Books Reviewed). The number of entries removed will be recorded. The remaining articles will be imported into the Rayyan software [37] (http://rayyan.qcri.org/) for title/abstract screening.

#### Assessing search comprehensiveness

Comprehensiveness of the search strategy will be assessed by: (1) determining whether all articles in a predetermined “test set” of approximately 15 relevant papers per stressor-response relationship (i.e., TN—chl-a, TP— chl-a; Table 3) are found with the search strategy; and (2) examining bibliographies of these “test set” papers, and papers that cite the “test set” papers, to determine whether relevant citations are captured in our search. If articles are missed, the search strategy will be evaluated and revised accordingly. The “test set” was created by searching the authors’ personal libraries for highly relevant articles until at least 15 papers per stressor-response relationship were obtained, and includes both journal articles and reports (Table 3).

### Article screening and study inclusion criteria

#### Screening process

Before screening all articles, consistency in applying inclusion criteria will be evaluated on a subset of articles using the kappa statistic (ranging from 0 to 1, with 1 indicating complete agreement [38]). Two to four reviewers will assess the same randomly-selected set of 10% of studies to be screened (minimum 50, maximum 200) at the title/abstract level. Kappa will be calculated, using modifications for more than two raters if necessary [39]. If kappa is low (<0.50) [40], reviewers will examine inconsistencies and clarify inclusion criteria; if kappa is moderate or high (>0.50) [40], one to four reviewers will proceed to screen all retrieved articles at the title/abstract level and, subsequently, all relevant articles at the full text level. Consistency during full text screening will be addressed by frequently convening reviewers to discuss the strategy and discuss and resolve any questions.

The inclusion criteria (see below) will be applied to systematically exclude articles that are topically irrelevant or do not contain relevant data, based on review of the title and abstract. Any article for which there is uncertainty about whether to include or exclude it based on title/abstract screening will be included for full text screening. Following evaluation of all titles and abstracts, full text screening will occur simultaneously with data extraction and quality assessment: as full text articles are examined for data extraction and quality assessment, any article judged to be irrelevant will be excluded and added to the appendix of excluded references, along with the justification based on inclusion criteria. Articles obtained through website searches will be screened during those searches by examining title/abstract/summary and full text when necessary, and information on the number of returns and relevant articles will be recorded separately.

#### Inclusion criteria

The following inclusion criteria will be used to determine relevant studies (see also Table 4):

Relevant population: Lotic freshwaters anywhere in the world or mesocosms made to mimic these systems.

Relevant exposure: Exposure to total nitrogen (TN) or total phosphorus (TP) measured as concentration (e.g. mg/L).

Relevant comparator: Comparison to sites or treatments with lower or higher levels of TN or TP across a gradient, or comparison to a control group (no or background TN or TP) or to lower or higher concentrations of TN or TP in experimental studies.

Relevant outcome: Concentration of benthic or ses-tonic chl-a, measured as mass per area or volume (e.g. μg/cm^2^, mg/m^2^, μg/L).

Relevant study type(s): Experimental studies in mesocosms or field sites, or field-based observational studies.

Relevant publication type(s): Study must contain original data and sufficient detail on methodology to assess study quality. Book chapters and conference abstracts will be excluded unless specifically suggested by outside experts.

Language: No language restrictions will be applied.

Date: No date restrictions will be applied.

#### Multiple studies using same datasets

For cases in which multiple studies use the same or similar datasets (e.g. a dissertation and one or more published articles from that dissertation), the following criteria (listed in order of priority) will be used to select a single source: the study with the more complete dataset, the version published as a peer-reviewed journal article, or the most recent version. The excluded duplicative study or studies may be used to fill in gaps in methodology or contextual information. These decisions will be documented in an appendix.

#### Unobtainable articles

Attempts to obtain full text of all articles not excluded during the screening process will be made using available library resources or by contacting authors. Articles for which full text is not obtainable will be listed in an appendix. Abstracts of non-English language articles will be translated using Google Translate to assess relevance. Every effort will be made to obtain translations of any highly relevant, non-English language papers; however, this will depend on available resources. All non-English articles considered relevant based on title/abstract screening but not fully translated will be listed in an appendix.

### Potential effect modifiers and reasons for heterogeneity

One motivation for this review is the apparent variability in nutrient stressor-response relationships in lotic ecosystems. Factors that potentially modify stressor-response relationships will be extracted from relevant studies when these factors were examined in the original study. Based on evaluation of highly relevant studies and consultation with stakeholders and experts, the modifiers considered include:

- ecoregion;
- latitude;
- altitude;
- land cover/land use;
- stream size;
- watershed area;
- geographic location;
- date/season/duration of sampling;
- stream gradient;
- flood stage/flow regime/flow permanence;
- nutrient concentration range (lowest and highest TN and/or TP);
- existing background nutrient concentrations;
- temperature;
- canopy cover/light availability;
- pH;
- alkalinity;
- sediment/turbidity;
- conductivity;
- dominant algal species/groups; and
- grazing (primary consumer) pressure.

Other relevant modifying factors will be recorded as they are encountered during screening and data extraction. Existing geographic information system (GIS) layers and tools that summarize important landscape and environmental factors (e.g. StreamCat [41], Google Earth) may be used to obtain relevant modifying factors (e.g. latitude, flow regime, land use/land cover, watershed area) for studies that do not report this information. If any outside data are associated with studies, care will be taken so as not to combine data from disparate sources (e.g. if the National Land Cover Dataset is used to estimate land cover, it will be used for all studies). Methodological modifiers, such as extraction method, measurement method, or sampling location (benthic, sestonic) for chl-a [31, 42], or fraction of water sample used for nutrient measurement (filtered, unfiltered), will also be recorded.

### Study quality assessment

Studies from articles included after title/abstract screening that are still categorized as relevant upon full text screening will be assessed for quality and risk of bias. Aspects of quality and risk of bias from published critical appraisal frameworks in environmental science and medicine [43–45] were examined to develop a quality assessment approach specific to this review, similar to [46] (Tables 5, 6 and 7). For each study, aspects of study quality contributing to a “low” or “high” risk of bias will be rated, based on specific criteria for three different study designs: (1) observational field studies, which typically sample chl-a along a gradient of nutrient concentrations; (2) mesocosm experiments; and (3) field experiments (e.g. Before-After-Control-Impact designs [47]) (Tables 5, 6 and 7). An overall risk of bias estimate for each study will be generated by dividing the number of “high” scores by the number of questions. Results of the systematic review will be discussed and analyzed in the context of this study quality assessment. All relevant studies will undergo quality assessment. To assess accuracy in quality assessment, a reviewer not involved in the initial quality assessment will independently assess quality for 25% of the studies evaluated by other reviewers, and reviewers will discuss and resolve any differences.

### Data extraction

Data will be extracted from studies found in articles that are considered relevant after full text screening. The majority of studies of nutrient stressor-response relationships examine biotic responses across field sites with varying nutrient concentrations, although some compare “reference” to “impacted” sites or experimentally manipulate nutrient concentrations. Most studies will thus use correlation or regression to assess relationships between nutrients and chl-a. The shape and direction (e.g. linear—increasing, linear—decreasing, logarithmic, exponential, sigmoidal) and strength of these relationships will form the basis for meta-analysis and narrative summary of the review results. In most instances, Pearson’s correlation coefficient or Spearman’s rho (r) between TN or TP and chl-a will be used as the effect size. Other effect size measures (e.g. standardized slope coefficients: change in standard deviations of y associated with a change of one standard deviation of x [47– 50]) will also be extracted and explored; however, the correlation coefficient was the most widely used and easily calculable from the example studies examined. Sample sizes will also be extracted for each effect size to estimate effect size variances using meta-analysis models (see “Data synthesis and presentation”). For experimental studies that manipulate nutrient concentrations and report differences in chl-a concentration between control and treatment groups, we will extract or calculate an appropriate “standardized mean difference” effect statistic such as Cohen’s *d* [50, 51].

Authors will be contacted if a study indicates that an effect size was calculated, but not reported (e.g. for negative associations). For studies not reporting effect sizes, raw data will be extracted from figures using image analysis software when possible and effect sizes will be calculated. If no effect size is reported and raw data are not presented (e.g. only site means are provided in a table), these studies will not be use in meta-analysis. The initial “test set” of relevant literature will be used to refine the data extraction fields as needed.

One to six reviewers will participate in data extraction from all relevant studies. To assess accuracy in data extraction, a reviewer not involved in initial data extraction will independently extract data for 25% of studies, and any differences will be discussed and resolved. Extracted data from relevant studies will be provided as an appendix or in a publicly-available USEPA data repository.

### Data synthesis and presentation

Meta-analysis and narrative and tabular summaries of stressor-response relationships will be used to synthesize data from the systematic review. For all studies, the direction or shape of the response will be noted (see “Data extraction”) and summarized across studies and subgroups of interest (e.g. subsets based on ecoregion, stream size, chl-a or nutrient measurement method). For studies with sufficient information, effect sizes (see “Data extraction”) and variance within and among studies will be examined across studies using a random effects model. Random effects models assume that the true effect size differs among studies and treat this heterogeneity as random, and are appropriate for making unconditional inferences about a set of studies of which the obtained studies are assumed to be a random sample [51–54]. Pearson’s correlation coefficient or Spearman’s rho (r) between TN or TP and chl-a will be used as the effect size in most instances. A Fisher’s z-transformation of r will likely be necessary to improve normality and variance [55, 56], although other effect size measures (e.g. standardized slope coefficients) will be explored. Equations in Nakagawa and Cuthill [50], Lajeunesse [51] and meta-analysis packages in the R environment [57] (e.g. ‘MAc’ [58] and citations therein) will be used to convert other effect sizes (e.g. multiple regression coefficients) to Pearson’s r. For analysis and presentation, results for TN and TP will be analyzed separately.

Effects of modifying factors (e.g. canopy cover) or subgroupings (e.g. ecoregion) will be assessed using mixed-effects models or meta-regression. Effect size variation and mean effect size will be visualized using forest plots. Analyses will be conducted using several R packages, including ‘metafor’ [53] and ‘MAc’ [58]. Quality assessment scores will be used as factors in sensitivity analysis to explore the impact of study quality on overall effect sizes and response shapes [40]. Publication bias will be assessed using funnel plots comparing study effect sizes with standard error [59, 60].

## Figures and Tables

**Table 1:** Search terms to be used for database searches

Habitat terms	Nutrient terms	Chlorophyll *a* terms
benth*	“total nitrogen”	chlorophyll
catchment	“total N”	“chlorophyll-a”
watershed		“chl-a”
stream*	“total phosphorus”	“chl a”
creek*	“total P”	
river*		
pool*		
“flood plain”		
floodplain		
riparia*		
ditch*		
lotic		
spring*		
seep*		
riffle*		
freshwater		
freshwaters		
“fresh water”		
brook		
“running water”		
headwater		
tributary		
mesocosm		
flume		
microcosm		

Terms within each of the three categories are combined with “OR” and the three categories are combined with “AND” for Boolean searches

**Table 2:** Bibliographic databases and relevant information

Database	Field to search	Publication types covered
ISI Web of Science Core Collection (Science Citation Index Expanded)	Topic	Peer-reviewed articles and conference proceedings
Scopus	Advanced search bar	Peer-reviewed articles and conference proceedings
JSTOR Life Sciences Archives	Full text—item type: articles	Peer-reviewed articles
CAB Abstracts and CAB Archive	Separate abstract and title searches	Peer-reviewed articles, books and conference proceedings
ProQuest Environmental Science Collection	Anywhere—except full text	Peer-reviewed and non-peer-reviewed articles, books, reports, and conference proceedings
BioOne	All fields	Peer-reviewed articles
Ingenta Connect	Title, keyword, abstract	Peer-reviewed articles
Science Direct	Title/abstract/keywords—(Boolean)	Peer-reviewed articles and books
Wiley Online Library	Separate searches for abstract and title	Peer-reviewed articles and books
OpenGrey	Default—treated as website for screening purposes due to download limitations	Unpublished reports, dissertations, conference papers, other grey literature
National Technical Reports Library	Advanced search—treated as website for screening purposes due to download limitations	Federally-funded technical reports
Greenfile	All fields	Published and unpublished papers and data
AGRICOLA	Articles search—advanced search “gkey”	Published and unpublished articles and data
AGRIS	Default search	Published and unpublished papers and data
ProQuest dissertations and theses A & I	All fields except full text	Dissertations and theses
DART	Default search—treated as website for screening purposes due to download limitations	Dissertations and theses

**Table 3:** “Test set” of sources used to test search strategy comprehensiveness and trial study quality and data extraction approaches

Citation	Year	TN	TP
Bourassa and Cattaneo [61]	1998	x	x
Braccia et al. [62]	2014	x	
Chambers et al. [32]	2012	x	x
DeNicola and Lellock [63]	2015	x	x
Heiskary and Bouchard [25]	2015		x
Justus et al. [64]	2010	x	x
Lewis and McCutchan [65]	2010		x
Lohman et al. [66]	1992	x	x
Maret et al. [15]	2010	x	x
Morgan et al. [67]	2006	x	x
Pan et al. [68]	1999	x	x
Stevenson et al. [69]	2006	x	x
Weigel and Robertson [70]	2007	x	x
Zheng et al. [71]	2008	x	x
Rier and Stevenson [72]	2006	x	x
Heiskary et al. [73]	2013	x	x
Iowa Department of Natural Resources [74]	2013	x	x

TN and TP columns indicate the nutrient(s) for which each citation reports a relationship with chl-a

**Table 4:** Detailed inclusion and exclusion criteria used to determine study inclusion in the systematic review

Inclusion criteria	Exclusion criteria
Population (unit of study)^a^	
-Lotic fresh waters anywhere in the world;	-Lentic or non-fresh waters (wetlands, lakes, reservoirs, ponds, oceans, estuaries)
-Mesocosms made to mimic lotic freshwater systems.	
Exposure (environmental variable to which population is exposed)	
-Exposure to total nitrogen (TN) or total phosphorus (TP) measured as concentration (e.g. mg/L)	-Exposure only to other nutrients, or nitrogen and phosphorus not reported as TN or TP
Comparators (control or alternative intervention)	
-Comparison to sites or treatments with lower or higher levels of TN or TP across a gradient;	-Studies of single sites (without sampling across time) or those without comparison to lower or higher levels of TN or TP.
-Comparison to control group (no or background TN or TP) or to lower or higher levels of TN or TP in experimental studies.	
Outcomes (relevant outcomes resulting from exposure)	
-Concentration of benthic or sestonic chlorophyll a, measured as mass per area or volume (e.g. μg/cm2, mg/m2, μg/L)	-Studies examining only TN or TP with no data on biological responses;
	-Studies examining other biological effects
Study type	
-Experimental studies in mesocosms or field sites;	-Studies examining only TN or TP with no data on biological responses;
-Field-based, observational studies	-Studies examining only biological effects other than chlorophyll *a*
Publications (types of sources used)	
-Study must contain original data;	-Articles with no original data (e.g. editorials, reviews);
-Study must contain sufficient detail on methodology to assess study quality	-Articles without sufficient information to evaluate pertinent relationships (chlorophyll a response to TN or TP) or study quality (e.g. methodology);
	-Retracted articles

a We included some search terms that may capture studies in lentic habitats related to flowing systems (e.g. floodplain, riparian) in an attempt to obtain relevant studies that might otherwise be missed. We recognize that there is some uncertainty with the lotic/lentic distinction (e.g. flowing freshwater springs) and will liberally include such articles at the title/abstract screening if otherwise relevant

**Table 5:** Study quality assessment framework for observational, field studies

Bias area	Characteristic	Low risk of bias	High risk of bias
Study design and sampling	Pairing of nutrient and chl-a measurements	Nutrient and chl-a measurements taken at same place and time or index period	Nutrient and chl-a measurements are not paired in time and space
	Study timeframe	Sampling includes relevant periods over multiple years	Sampling occurs only over a single season or year
	Gradient definition	Gradient based on a nutrient related variable or its causal antecedent	Gradient based on a common causal descendant of TP,TN or chl-a
	Sample size	Acceptable # sites (≥10) across gradient	Low # sites (≥10) across gradient low
	Replicates	Multiple samples taken at each site	Single samples taken at each site
	Randomization of sampling (selection bias)	Some form of randomized site selection (e.g. stratified random sampling)	No randomization of site selection
	Confounding factors	If not controlled by study design, confounding factors are measured and adjusted for in statistical analysis	Confounding factors reported and not accounted for, or are likely, and are not able to be adjusted for post hoc
Data analysis and results	Clarity and detail	Analysis methods described in detail sufficient to permit repeating	Missing information not allowing for repeatability
	Uncertainty	Some estimate of uncertainty in effect or relationships provided (e.g. confidence intervals, standard error, standard deviation, etc.)	No estimates of uncertainty provided
	Reporting bias	All variables, measurements, and statistical tests mentioned in methods are reported in results or additional file	Some variables, measurements, or statistical tests mentioned in methods are not reported
Other biases	Detection bias	No indication that outcomes were measured differently in high versus low exposure sites	Some indication that outcomes were measured differently in high versus low exposure sites
	Attrition bias	No differences in loss of high versus low exposure sites	Differences in loss of high versus low exposure sites
	Research aim consistency	Questions clearly stated and answers match questions	Questions not clearly stated or answers do not match stated questions

**Table 6:** Study quality assessment framework for experimental mesocosm studies

Bias area	Characteristic	Low risk of bias	High risk of bias
Study design and sampling	Study timeframe	Study timeframe considers risk of container effects (<30 days, but can depend on size and flow-through vs. recirculating)	Study timeframe is long enough to be at risk of container effects and other random processes (typically >30 days but depends on size and flow-through vs. recirculating)
	Randomization of sampling (selection bias)	Some form of randomized assignment of control and treatment samples	No randomization of control and treatment assignments
	Control matching and performance bias	Control and treatment mesocosms are similar, or there is a clear effort to account for any differences	Evidence that control and treatment mesocosms differ in aspects not related to treatments, with no effort to account for differences
	Treatment clarity and detail	Method of nutrient addition clearly explained (e.g. pulse, continuous drip)	Method of nutrient addition not clear
	Confounding factors	No obvious confounding factors reported, or if reported are accounted for	Confounding factors reported and not accounted for, unclear how accounted for, or are likely
	Replication	≥3 replicates per treatment, no pseudoreplication	<3 replicates per treatment, or evidence of pseudoreplication
	Measurement clarity and detail	Methods for design and sampling described in detail, including chl-a extraction and measurement, water filtering, and nutrient measurement	Missing information not allowing for repeatability
Data analysis and results	Clarity and detail	Analysis methods described in detail sufficient to permit repeating	Missing information not allowing for repeatability
	Uncertainty	Some estimate of uncertainty in effect or relationships provided (e.g. confidence intervals, standard error, standard deviation, etc.)	No estimates of uncertainty provided
	Treatment vs. control	Differences in treatments vs. controls reported quantitatively (e.g. actual values, response ratios, effect size)	Differences in treatments vs. controls reported only qualitatively (higher, lower) or otherwise unclear (e.g. only *P* value given)
	Reporting bias	All variables, measurements, and statistical tests mentioned in methods are reported in results or additional file	Some variables, measurements, or statistical tests mentioned in methods are not reported
Other biases	Detection bias	No indication that outcomes were measured differently in control and treatment samples	Some indication that outcomes were measured differently in control and treatment samples
	Attrition bias	No differences in loss of control and treatment samples	Differences in loss of control and treatment samples
	Research aim consistency	Questions clearly stated and answers match questions	Questions not clearly stated or answers do not match stated questions

**Table 7:** Study quality assessment framework for experimental field studies

Bias area	Characteristic	Low risk of bias	High risk of bias
Study design and sampling	Study type	Before-After-Control-1 mpact design	Before-After or Control-1 mpact design
	Treatment replication	Treatment is applied or studied at multiple independent sites	Treatment not replicated at multiple sites
	Study timeframe	Sampling occurs over multiple years	Sampling occurs only over a single season or year
	Pairing of nutrient and chl-a measurements	Nutrient and chl-a measurements are taken at the same place and time or index period	Nutrient and chl-a measurements are not paired in time and space
	Control matching and performance bias	Control and treatment systems are similar and there is a clear effort to account for any differences	Evidence that control and treatment systems differ in aspects not related to treatments, with no effort to account for differences
	Treatment clarity and detail	Method of nutrient addition clearly explained (e.g. pulse, continuous drip)	Method of nutrient addition not clear
	Confounding factors	Sample design minimizes effect of obvious confounding factors; or if not controlled by sample design, they are measured and adjusted for in statistical analysis	Confounding factors reported and not accounted for, or are likely, and are not able to be adjusted for post hoc
	Temporal replication	>1 replicate before and after treatment	1 replicate before and after treatment
	Spatial replication	>1 site per control/impact	1 site per control/impact
	Measurement clarity and detail	Methods for design and sampling described in detail, including chl-a extraction and measurement, water filtering, and nutrient measurement	Missing information not allowing for repeatability
Data analysis and results	Clarity and detail	Analysis methods described in detail sufficient to permit repeating	Missing information not allowing for repeatability
	Uncertainty	Some estimate of uncertainty in effect or relationships provided (e.g. confidence intervals, standard error, standard deviation, etc.)	No estimates of uncertainty provided
	Treatment vs. Control	Differences in treatments vs. controls reported quantitatively (e.g. actual values, response ratios, effect size)	Differences in treatments vs. controls reported only qualitatively (higher, lower) or are otherwise unclear (e.g. only P value given)
	Reporting bias	All variables, measurements, and statistical tests mentioned in methods are reported in results or additional file	Some variables, measurements, or statistical tests mentioned in methods are not reported
Other biases	Detection bias	No indication that outcomes were measured differently in control and treatment samples	Some indication that outcomes were measured differently in control and treatment samples
	Attrition bias	No differences in loss of control and treatment samples	Differences in loss of control and treatment samples
	Research aim consistency	Questions clearly stated and answers match questions	Questions not clearly stated or answers do not match stated questions
	Other bias	No evidence of other sources of bias	Evidence of bias from a source not considered above

## References

[1] Dubrovsky NM, Hamilton P. Nutrients in the Nation’s streams and groundwater: National Findings and Implications. Nattional Water-Quality Assessment Program. Fact Sheet, United States Geological Survey 2010.

[2] Cao D, Cao W, Fang J, Cai L. Nitrogen and phosphorus losses from agricultural systems in China: a meta-analysis. Mar Pollut Bull 2014;85:727–32. 24934439 10.1016/j.marpolbul.2014.05.041

[3] Jarvie HP, Sharpley AN, Spears B, Buda AR, May L, Kleinman PJ. Water quality remediation faces unprecedented challenges from “legacy phosphorus”. Environ Sci Technol 2013;47:8997–8. 23931665 10.1021/es403160a

[4] Smith V. Eutrophication of freshwater and coastal marine ecosystems a global problem. Environ Sci Pollut Res 2003;10:126–39. 10.1065/espr2002.12.142 12729046

[5] Conley DJ, Paerl HW, Howarth RW, Boesch DF, Seitzinger SP, Havens KE, Controlling eutrophication: nitrogen and phosphorus. Science 2009;323:1014–5. 19229022 10.1126/science.1167755

[6] Compton JE, Harrison JA, Dennis RL, Greaver TL, Hill BH, Jordan SJ, Ecosystem services altered by human changes in the nitrogen cycle: a new perspective for US decision making. Ecol Lett 2011;14:804–15. 21624028 10.1111/j.1461-0248.2011.01631.x

[7] Dodds WK, Bouska WW, Eitzmann JL, Pilger TJ, Pitts KL, Riley AJ, Eutrophication of US freshwaters: analysis of potential economic damages. Environ Sci Technol 2009;43:12–9. 19209578 10.1021/es801217q

[8] Beauvais J. Renewed call to action to reduce nutrient pollution and support for incremental actions to protect water quality and public health Washington, DC: U.S Environmental Protection Agency; 2016 p. 6 https://www.epa.gov/sites/production/files/2016-09/documents/renewed-call-nutrient-memo-2016.pdf. Accessed 15 June 2017.

[9] Stoner N. Working in partnership with states to address phosphorus and nitrogen pollution through use of a framework for state nutrient reductions Washington, DC: U.S. Environmental Protection Agency; 2011 p. 6.

[10] Dodds WK, Smith VH, Lohman K. Nitrogen and phosphorus relationships to benthic algal biomass in temperate streams. Can J Fish Aquat Sci 2002;59:865–74.

[11] Rosemond AD, Benstead JP, Bumpers PM, Gulis V, Kominoski JS, Manning DWP, Experimental nutrient additions accelerate terrestrial carbon loss from stream ecosystems. Science 2015;384:318–21. 10.1126/science.aaa1958 25745171

[12] Dodds WK, Smith VH. Nitrogen, phosphorus, and eutrophication in streams. Inland Waters 2016;6:155–64.

[13] Biggs BJF. Eutrophication of streams and rivers: dissolved nutrient-chlorophyll relationships for benthic algae. J N Am Benthol Soc 2000;19:17–31.

[14] Biggs BJ. The contribution of flood disturbance, catchment geology and land use to the habitat template of periphyton in stream ecosystems. Freshw Biol 1995;33:419–38.

[15] Maret TR, Konrad CP, Tranmer AW. Influence of environmental factors on biotic responses to nutrient enrichment in agricultural streams. J Am Water Resour Assoc 2010;46:498–513. 22457568 10.1111/j.1752-1688.2010.00430.x PMC3307628

[16] Van Meter KJ, Basu NB, Veenstra JJ, Burras CL. The nitrogen legacy: emerging evidence of nitrogen accumulation in anthropogenic landscapes. Environ Res Lett 2016;11:1–12.

[17] Sharpley A, Jarvie HP, Buda A, May L, Spears B, Kleinman P. Phosphorus legacy: overcoming the effects of past management practices to mitigate future water quality impairment. J Environ Qual 2013;42:1308. 24216410 10.2134/jeq2013.03.0098

[18] Pellerin BA, Bergamaschi BA, Gilliom RJ, Crawford CG, Saraceno J, Frederick CP, Mississippi River nitrate loads from high frequency sensor measurements and regression-based load estimation. Environ Sci Technol 2014;48:12612–9. 25310505 10.1021/es504029c

[19] Poff NL, Ward JV. Physical habitat template of lotic systems: recovery in the context of historical pattern of spatiotemporal heterogeneity. Environ Manag 1990;14:629–45.

[20] Smith VH. Effects of eutrophication on maximum algal biomass in lake and river ecosystems. Inland Waters 2016;6:147–54.

[21] Barbour MT, Gerritsen J, Snyder BD, Stribling JB. Rapid bioassessment protocols for use in streams and wadeable rivers: periphyton, benthic macroinvertebrates and fish 2nd ed. Washington, DC: U.S Environmental Protection Agency Offices Water 1998 https://archive.epa.gov/water/archive/web/html/index-14.html. Accessed 15 June 2017.

[22] Nichols SJ, Barmuta LA, Chessman BC, Davies PE, Dyer FJ, Harrison ET, The imperative need for nationally coordinated bioassessment of rivers and streams. Mar Freshw Res 2017;68:599–613.

[23] Hering D, Borja A, Carstensen J, Carvalho L, Elliott M, Feld CK, The European Water Framework Directive at the age of 10: a critical review of the achievements with recommendations for the future. Sci Total Environ 2010;408:4007–19. 20557924 10.1016/j.scitotenv.2010.05.031

[24] Karr JR. Defining and measuring river health. Freshw Biol 1999;41:221–34.

[25] Heiskary SA, Bouchard RW, Heiskary SA. Development of eutrophication criteria for Minnesota streams and rivers using multiple lines of evidence. Freshw Sci 2015;34:574–92.

[26] Dodds WK, Welch EB. Establishing nutrient criteria in streams. J N Am Benthol Soc 2000;19:186–96.

[27] Sutula M. Review of indicators for development of nutrient numeric endpoints in California estuaries. Costa Mesa: Southern California Coastal Water Research Project; 2011.

[28] Dodds WK. Trophic state, eutrophication and nutrient criteria in streams. Trends Ecol Evol 2007;22:669–76. 17981358 10.1016/j.tree.2007.07.010

[29] Smith DR, King KW, Williams MR. What is causing the harmful algal blooms in Lake Erie? J Soil Water Conserv 2015;70:27A–9A.

[30] Otten TG, Paerl HW. Health effects of toxic cyanobacteria in U.S. drinking and recreational waters: our current understanding and proposed direction. Curr Environ Heal Rep 2015;70:75–84. 10.1007/s40572-014-0041-9 26231244

[31] Steinman AD, Lamberti GA, Leavitt PR. Biomass and pigments of benthic algae In: Hauer FR, Lamberti GE, editors. Methods in stream ecology Burlington: Academic Press; 2006 p. 357–79.

[32] Chambers PA, McGoldrick DJ, Brua RB, Vis C, Culp JM, Benoy GA. Development of environmental thresholds for nitrogen and phosphorus in streams. J Environ Qual 2012;41:7–20. 22218169 10.2134/jeq2010.0273

[33] Dodds WK. What controls levels of dissolved phosphate and ammonium in surface waters? Aquat Sci 1993;55:132–42.

[34] Dodds WK, Smith VH, Zander B. Developing nutrient targets to control benthic chlorophyll levels in streams: a case study of the Clark Fork River. Water Res 1997;31:1738–50.

[35] Dodds WK. Misuse of inorganic N and soluble reactive P concentrations to indicate nutrient status of surface waters. J N Am Benthol Soc 2003;22:171–81.

[36] Bennett MG, Lee SS. Lotic ecosystem responses to nutrients: a research-to-policy gap in experimental studies. Environ Sci Technol Submitt

[37] Ouzzani M, Hammady H, Fedorowicz Z, Elmagarmid A. Rayyan—a web and mobile app for systematic reviews. Syst Rev 2016;5:210. 27919275 10.1186/s13643-016-0384-4 PMC5139140

[38] Cohen J. A coefficient of agreement for nominal scales. Educ Psychol Meas 1960;20:37–46.

[39] Fleiss JL. Measuring nominal scale agreement among many raters. Psychol Bull 1971;76:378–82.

[40] Collaboration for environmental evidence. Guidelines for systematic review and evidence synthesis in environmental management. Version 4.2 2013 http://www.environmentalevidence.org/wp-content/uploads/2014/06/Review-guidelines-version-4.2-final.pdf. Accessed 15 June 2017.

[41] Hill RA, Weber MH, Leibowitz SG, Olsen AR, Thornbrugh DJ. The Stream-Catchment (StreamCat) dataset : a database of watershed metrics for the coterminous United States. JAWRA J Am Water Resour Assoc 2016;52:120–8.

[42] Hagerthey SE, William Louda J, Mongkronsri P. Evaluation of pigment extraction methods and a recommended protocol for periphyton chlorophyll a determination and chemotaxonomic assessment. J Phycol 2006;42:1125–36.

[43] Mupepele A-C, Walsh JC, Sutherland WJ, Dormann CF. An evidence assessment tool for ecosystem services and conservation studies. bioRxiv Prep 2015;26:1295–301. 10.1890/15-0595 27755765

[44] Bilotta GS, Milner AM, Boyd IL. Quality assessment tools for evidence from environmental science. Environ Evid 2014;3:14.

[45] Higgins J, Green S, editors. Cochrane handbook for systematic reviews of interventions Version 5. The Cochrane Collaboration; 2011 http://www.handbook.cochrane.org. Accessed 15 June 2017.

[46] Haddaway NR, Burden A, Evans CD, Healey JR, Jones DL, Dalrymple SE, Evaluating effects of land management on greenhouse gas fluxes and carbon balances in boreo-temperate lowland peat systems. Environ. Evid 2014;3:5.

[47] Underwood AJ. Beyond BACI—the detection of environmental impacts on poulations in the real, but variable, world. J Exp Mar Biol Ecol 1992;161:145–78.

[48] Te Morenga L, Mallard S, Mann J. Dietary sugars and body weight: systematic review and meta-analyses of randomised controlled trials and cohort studies. BMJ 2013;346:e7492. 10.1136/bmj.e7492 23321486

[49] Johnson PI, Sutton P, Atchley DS, Koustas E, Lam J, Sen S. The navigation guide—evidence-based medicine meets environmental health: systematic review of human evidence for PFOA effects on fetal growth. Environ Heal Perspect 2014;122:1028–39. 10.1289/ehp.1307893 PMC4181929 24968388

[50] Nakagawa S, Cuthill IC. Effect size, confidence interval and statistical significance: a practical guide for biologists. Biol Rev 2007;82:591–605. 17944619 10.1111/j.1469-185X.2007.00027.x

[51] Lajeunesse M. Recovering missing or partial data from studies: a survey of conversions and imputations for meta-analysis In: Gurevitch J, Mengersen K, editors. Handbook of meta-analysis ecology evolution Princeton: Princeton University Press; 2013 p. 195–206.

[52] Hedges LV, Vevea JL. Fixed- and random-effects models in meta-analysis. Psychol Methods 1998;3:486–504.

[53] Viechtbauer W. Conducting meta-analyses in R with the meta for package. J Stat Softw 2010;36:1–48.

[54] Gurevitch J, Hedges LV. Statistical issues in ecological meta-analysis. Ecology 1999;80:1142–9.

[55] Worm B, Myers RA. Meta-analysis of cod-shrimp interactions revels top-down control in ocean food webs. Ecology 2003;84:162–73.

[56] Lassauce A, Paillet Y, Jactel H, Bouget C. Deadwood as a surrogate for forest biodiversity: meta-analysis of correlations between deadwood volume and species richness of saproxylic organisms. Ecol Indic 2011;11:1027–39.

[57] R Core Team. R: a language and environment for statistical computing. Vienna, Austria: R Foundation for Statistical Computing; 2016 https://www.r-project.org. Accessed 15 June 2017.

[58] Del Re A, Hoyt WT. Package “MAc”: meta-analysis with correlations 2015 https://cran.r-project.org/web/packages/MAc/MAc.pdf. Accessed 15 June 2017.

[59] Palmer AR. Datecting publication bias in meta-analyses: a case study of fluctuating asymmetry and sexual selection. Am Nat 1999;154:220–33. 29578786 10.1086/303223

[60] Egger M, Davey Smith G, Schneider M, Minder C. Bias in meta-analysis detected by a simple, graphical test. BMJ 1997;315:629–34. 9310563 10.1136/bmj.315.7109.629 PMC2127453

[61] Bourassa N, Cattaneo A. Control of periphyton biomass in Laurentian streams (Québec). J N Am Benthol Soc 1998;17:420–9.

[62] Braccia A, Eggert SL, King N. Macroinvertebrate colonization dynamics on artificial substrates along an algal resource gradient. Hydrobiologia 2014;727:1–18.

[63] DeNicola DM, Lellock AJ. Nutrient limitation of algal periphyton in streams along an acid mine drainage gradient. J Phycol 2015;51:739–49. 26986794 10.1111/jpy.12315

[64] Justus BG, Petersen JC, Femmer SR, Davis JV, Wallace JE. A comparison of algal, macroinvertebrate, and fish assemblage indices for assessing low-level nutrient enrichment in wadeable Ozark streams. Ecol Indic 2010;10:627–38.

[65] Lewis WM, Mccutchan JH. Ecological responses to nutrients in streams and rivers of the Colorado mountains and foothills. Freshwater Biology 2010;55:1973–83.

[66] Lohman K, Jones JR, Perkins BD. Effects of nutrient enrichment and flood frequency on periphyton biomass in northern Ozark streams. Can J Fish Aquat Sci 1992;49:1198–205.

[67] Morgan AM, Royer TV, David MB, Gentry LE. Relationships among nutrients, chlorophyll-a, and dissolved oxygen in agricultural streams in Illinois. J Environ Qual 2006;35:1110–7. 16738396 10.2134/jeq2005.0433

[68] Pan Y, Stevenson RJ, Hill BH, Kaufmann PR, Herlihy AT. Spatial patterns and ecological determinants of benthic algal assemblages in mid-Atlantic streams, USA. J Phycol 1999;35:460–8.

[69] Stevenson RJ, Rier ST, Riseng CM, Schultz RE, Wiley MJ. Comparing effects of nutrients on algal biomass in streams in two regions with different disturbance regimes and with applications for developing nutrient criteria. Hydrobiologia 2006;561:149–65.

[70] Weigel BM, Robertson DM. Identifying biotic integrity and water chemistry relations in nonwadeable rivers of Wisconsin: toward the development of nutrient criteria. Environ Manag 2007;40:691–708. 10.1007/s00267-006-0452-y 17638041

[71] Zheng L, Gerritsen J, Beckman J, Ludwig J, Wilkes S. Land use, geology, enrichment, and stream biota in the Eastern Ridge and Valley ecoregion: implications for nutrient criteria development. J Am Water Resour Assoc 2008;44:1521–36.

[72] Rier ST, Stevenson RJ. Response of periphytic algae to gradients in nitrogen and phosphorus in streamside mesocosms. Hydrobiologia 2006;561:131–47.

[73] Heiskary SA, Bouchard RW, Markus H. Minnesota nutrient criteria development for rivers. Saint Paul: Minnesota Pollution Control Agency; 2013.

[74] Iowa Department of Natural Resources Development of nutrient enrichment criteria for Iowa streams Des Moines: Iowa Department of Natural Resources; 2013.

